# Negative effects of an allelopathic invader on AM fungal plant species drive community‐level responses

**DOI:** 10.1002/ecy.3201

**Published:** 2020-11-04

**Authors:** Morgan D. Roche, Ian S. Pearse, Lalasia Bialic‐Murphy, Stephanie N. Kivlin, Helen R. Sofaer, Susan Kalisz

**Affiliations:** ^1^ Department of Ecology and Evolutionary Biology University of Tennessee Knoxville Tennessee 37996 USA; ^2^ U.S. Geological Survey Fort Collins Science Center Fort Collins Colorado 80526 USA; ^3^ Present address: U.S. Geological Survey Pacific Island Ecosystems Research Center Volcano Hawaii 96718 USA

**Keywords:** allelopathy, *Alliariapetiolata*, arbuscular mycorrhizal fungi, community change, garlic mustard, invasion impacts, long‐term experiment, mutualism disruption, symbiosis

## Abstract

The mechanisms causing invasive species impact are rarely empirically tested, limiting our ability to understand and predict subsequent changes in invaded plant communities. Invader disruption of native mutualistic interactions is a mechanism expected to have negative effects on native plant species. Specifically, disruption of native plant‐fungal mutualisms may provide non‐mycorrhizal plant invaders an advantage over mycorrhizal native plants. Invasive *Alliaria petiolata* (garlic mustard) produces secondary chemicals toxic to soil microorganisms including mycorrhizal fungi, and is known to induce physiological stress and reduce population growth rates of native forest understory plant species. Here, we report on a 11‐yr manipulative field experiment in replicated forest plots testing if the effects of removal of garlic mustard on the plant community support the mutualism disruption hypothesis within the entire understory herbaceous community. We compare community responses for two functional groups: the mycorrhizal vs. the non‐mycorrhizal plant communities. Our results show that garlic mustard weeding alters the community composition, decreases community evenness, and increases the abundance of understory herbs that associate with mycorrhizal fungi. Conversely, garlic mustard has no significant effects on the non‐mycorrhizal plant community. Consistent with the mutualism disruption hypothesis, our results demonstrate that allelochemical producing invaders modify the plant community by disproportionately impacting mycorrhizal plant species. We also demonstrate the importance of incorporating causal mechanisms of biological invasion to elucidate patterns and predict community‐level responses.

## Introduction

Biological invasions threaten biodiversity, with broad impacts on the economy, environment, health, and culture. Global meta‐analyses demonstrate that invasive species can have a range of negative impacts that include lower abundance and fitness of native species and altered microbial activity and nutrient availability in invaded sites (Vilà et al. 2011, Pyšek et al. 2012). Despite these observed negative consequences, we are still largely unable to predict the potential outcomes of an introduced species prior to its establishment and spread (Ricciardi et al. 2013, Sofaer et al. 2018). Since the impacts of invasion depend on the interaction of traits of individual invasive species (Gurevitch and Padilla 2004) and traits of the invaded community (Hejda et al. 2009), it is unlikely that a single mechanism will fully explain the impacts of invasion. However, understanding causal mechanisms by which certain types of invasive species exert impacts on species in the resident community can lead to more accurate predictions of where and when negative effects will occur in the invaded range (Levine et al. 2003).

Many studies have analyzed spatiotemporal patterns of community change to assess the impacts of an invasive species, but knowledge of the mechanism of impact is indispensable for generalizing individual or species‐level effects to those expected at the community level. Studies of community‐level patterns show that invasions can lead to biotic homogenization across locations (Sax et al. 2002) but rarely reduce local species richness within one location (Powell et al. 2011). In addition, invasive species alter community evenness by disproportionately displacing dominant native species (Powell et al. 2013, Pearse et al. 2019). Yet such patterns do not generally predict which species within the resident community will be most vulnerable to the invader, and thereby reliably link invader impacts at the individual, population, and community levels. In contrast, by understanding mechanisms of impact, we may be able to predict responses across levels of biological organization. For example, by understanding how herbivorous insects identify their host plants, we can anticipate the species‐specific impacts of invasive plants or insects on other trophic levels in the native food web (Pearse and Altermatt 2013, Desurmont and Pearse 2014). Moreover, evolutionary divergence time between native range host tree and invaded range host trees is a strong predictor of high impacts of introduced insects on forests (Mech et al. 2019). For trophic interactions (such as herbivory, above), the mechanism of impact is a function of diet, and can be relatively straightforward and consistent across space and time.

The indirect or competitive interactions that are typical of plant invaders are particularly difficult to detect and predict (Suding et al. 2008), and understanding mechanisms of invasion involving these types of interactions typically requires manipulative studies. Many processes can underlie invader impact (Ricciardi et al. 2013), but the mutualism disruption hypothesis (Hale and Kalisz 2012) is particularly well suited to evaluating invader impact from individual species to the community because we can make predictions about which native species within a community will be affected based on their known mutualism dependence. Under this hypothesis, an invasive species can gain a competitive advantage over mutualism‐dependent native species by reducing or eliminating the benefits of these mutualisms (Hale and Kalisz 2012, Traveset and Richardson 2014). The effects of mutualism disruption have been observed in a variety of invaded systems. Compelling examples include the disruption of ant seed dispersal in the presence of the Argentine ant (*Linepithema humile*) that resulted in a shift of the resident plant community based on seed and elaiosome size (Christian 2001, Rowles and Dowd 2009), the predation of seed dispersing birds to near extinction by the brown tree snake (*Boiga irregularis*) that severely reduced native tree recruitment on Guam (Rogers et al. 2017), and the usurpation of native pollination services by the showy flowered purple loosestrife (*Lythrum salicaria*) that led to a steep decline in pollinator visitation to native flowers (Brown et al. 2002). Mutualism disruption is widespread, and can negatively affect dispersal, reproductive, or nutritive mutualisms.

The global threat of mutualism disruption is particularly substantial for plant–mycorrhizal mutualisms because of their ubiquity (Soudzilovskaia et al. 2020) and their importance for plant growth, function, and community dynamics (Van Der Heijden et al. 2008). Invasive plants can impact plant–mycorrhizal mutualisms in various ways (Grove et al. 2017): by directly competing with native plants, which weakens the native plant’s contribution to their mycorrhizal partnership (Grove et al. 2017), by changing soil properties to alter the balance of resource exchange between plant and fungal partners (Allison et al. 2006, Johnson 2010), or by directly inhibiting the mutualism via invader‐produced toxic secondary chemistry (allelochemicals) (Hale and Kalisz 2012). The specific mechanism of mycorrhizal mutualism disruption may vary among invasive species but toxic allelochemistry is common. Using published lists of invasive plant families (Pyšek et al. 2012) and plant families with known allelopathic properties (Hale and Kalisz 2012), we estimate that 41% of invasive plant families are allelopathic (*unpublished data*). Of these allelopathic plant invaders, *Alliaria petiolata* (Brassicaceae, Bieb. Cavara & Grande, hereafter garlic mustard) is emerging as a model for understanding allelopathic mycorrhizal mutualism disruption (Cipollini and Cipollini 2016).

Experimental evidence in both controlled and field settings has established that garlic mustard reduces native plant performance and population growth through the disruption of their mycorrhizal associations (e.g., Stinson et al. 2006, Callaway et al. 2008, Wolfe et al. 2008, Barto et al. 2011, Hale et al. 2011, 2016, Brouwer et al. 2015, Bialic‐Murphy et al. 2019). Furthermore, garlic mustard is a poor direct competitor (Meekins and McCarthy 1999, Bossdorf et al. 2004), and has no known direct phytotoxic effects (Brouwer et al. 2015, Hale et al. 2016). In addition, there is evidence that an increase in garlic mustard abundance is associated with lower native plant diversity (Stinson et al. 2007), and that community‐level responses to garlic mustard vary by species and by abiotic conditions (Haines et al. 2018). However, it is unknown whether the physiological consequences of mutualism disruption for individual plant species, mediated by declines in function of plant–mycorrhizal fungal partnerships, are linked to the shifts in plant community diversity and composition following garlic mustard invasion (Stinson et al. 2007, Callaway et al. 2008, Cipollini and Cipollini 2016, McCary et al. 2019).

Here, we exploit the power of this well‐established mechanism of invasion impact to test for a link between mutualism disruption of mycorrhizal plant individuals and community‐level response of the forest understory to garlic mustard invasion. A large portion of the diversity in most Eastern deciduous forests exists in the understory, and these species are also diverse in their mycorrhizal associations (Gilliam 2007, Soudzilovskaia et al. 2020), making the forest understory community a highly relevant study system. We present analyses of data collected from a 11‐yr garlic mustard weeding experiment in replicated plots to assess whether garlic mustard differentially impacts native mycorrhizal vs. non‐mycorrhizal herbaceous understory plant species. We ask two questions: (1) Does the release and recovery from garlic mustard invasion (i.e., garlic mustard weeded treatment) differentially affect the community composition and diversity of mycorrhizal and non‐mycorrhizal herbaceous understory plant communities? (2) Are changes in community composition and diversity explained by shifts in abundance of mycorrhizal and non‐mycorrhizal plant species? Based on the mutualism disruption hypothesis, we expect a greater change in the community composition, diversity, and abundance of mycorrhizal plants (i.e., the functional groups most sensitive to garlic mustard’s allelochemicals) than non‐mycorrhizal plants, culminating in a distinct signature of mutualism disruption at the community level.

## Methods

### Study system

Trillium Trail Nature Reserve in Fox Chapel, Pennsylvania, USA (50.520237° N, 79.900932° W) is a beech–maple forest with a diverse understory plant community (Hale et al. 2011). The majority (~73%) of understory herbs at Trillium Trail partner with arbuscular mycorrhizal (AM) fungi (Hale et al. 2011) and provide photosynthetically derived carbon compounds in exchange for water and nutrients acquired by the fungi (Augé 2001, Van Der Heijden et al. 2008). Many of these mycorrhizal plant species have coarse roots with no root hairs (Brundrett and Kendrick 1988), which suggests that they rely heavily on their fungal partners for soil resources. For these reasons, we expect that the effects of mycorrhizal mutualism disruption by allelopathic plant invaders could be dramatic at Trillium Trail, making it an ideal location to test for evidence of mutualism disruption at the community level.

Garlic mustard, a biennial herb, is invasive to temperate forests of North America and colonizes forest understories (Anderson et al. 1996, Nuzzo 1999). Garlic mustard’s allelochemicals are toxic to AM fungi (Cantor et al. 2011). Exposure to these allelochemicals decreases growth and abundance of AM fungal hyphae in soil and roots (Rodgers et al. 2008, Cantor et al. 2011, Hale et al. 2011, 2016, Poon and Maherali 2015). As a result, native plants that rely on AM fungi for nutrient and water acquisition are negatively affected. Garlic mustard was first documented at Trillium Trail in the early 1990s (Knight et al. 2009). *Maianthemum racemosum*, a common species at Trillium Trail with coarse roots and high root AM fungal colonization rates, exhibited declines in carbon fixation and shifts in carbon storage, and altered vital rates following disruption of the mycorrhizal mutualism by garlic mustard (Brouwer et al. 2015, Hale et al. 2016). For another common mycorrhizal species at our site, *Trillium erectum*, garlic mustard invasion altered plant vital rates and reduced the population growth rate (Bialic‐Murphy et al. 2019). It is clear from these prior studies that garlic mustard disrupts the plant root AM fungal mutualism, the consequences of mutualism disruption are physiological in nature, and the physiological effects of mutualism disruption are strong enough to reduce population growth rates.

**Fig. 1:**
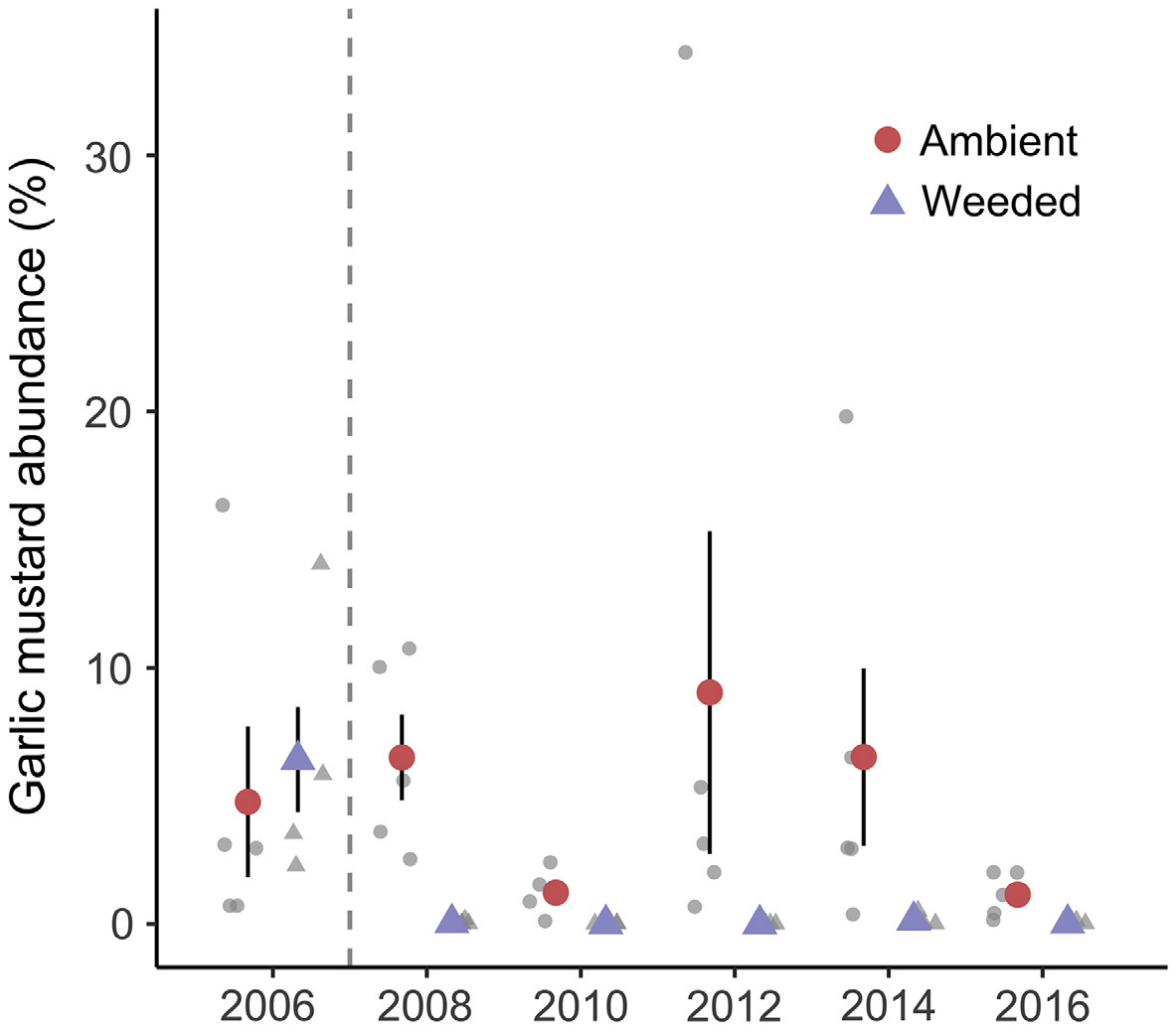
Graphical representation of garlic mustard treatment efficacy. Values are garlic mustard abundance (measured as percent cover; mean ± SE) in ambient (red circles) and weeded (purple triangles) treatments (2006–2016). Jittered data points for each plot in gray. The first year of garlic mustard weeding occurred after data collection in 2006. Vertical gray dashed line separates pre‐treatment (2006) from post‐treatment abundance values.

In 2006, we began an experimental treatment in five 14 × 14 m replicate plots that were surrounded by 2.5 m tall wire fencing (erected in 2002) to prevent deer access. Each plot was divided into 36, 2 × 2 m subplots. After sampling in 2006, the 18 subplots on the left half of each plot were weeded of all garlic mustard plants and the right half of each plot was not manipulated and remained at ambient levels of garlic mustard invasion. All garlic mustard individuals, including roots, were hand pulled from the weeded side of each plot, bagged, and disposed of off‐site. In every subsequent year, weeding occurred in the spring when garlic mustard germinates and all weeded material was removed from the site. To preclude garlic mustard seed dispersal into the weeded half of each plot, we installed temporary barriers annually just prior to garlic mustard seed dispersal that we removed when seed dispersal was complete. Persistent weeding was an effective method for removing the invader. Garlic mustard relative abundance in ambient plots averaged 15.2% and ranged from 0.5% to 71.6%. After weeding in 2006, garlic mustard abundance averaged 0.08% in the weeded plots due to infrequent late emerging seedlings (Fig. 1). These seedlings were subsequently removed.

In late June to early July in even years beginning in 2006, all herbaceous species within each subplot were identified to the species‐ or genus‐level. Since some species within a genus are not distinguishable from each other when they are not flowering (e.g., *Impatiens*,*Trillium*), these species were grouped by genus when estimating abundance. To estimate species abundance, two observers were trained using standardized percent cover area templates. Within a 2 × 2 m subplot, total leaf area occupied by each species was independently estimated by each observer, and we averaged estimated cover of the two observers. Total abundance across all species for any subplot could be> 100% due to leaf overlap. We calculated the mean abundance of each species across the 18 subplots for the garlic mustard weeded and garlic mustard ambient treatments within each plot in each sampling year.

### Data analyses

We assigned a mycorrhizal status (mycorrhizal or non‐mycorrhizal) to each recorded plant species based on available published and unpublished data (Appendix S1: Table S1 and citations therein). Species that form associations with AM fungi were assigned to the “mycorrhizal” functional group and species that do not form mycorrhizal associations were assigned to the “non‐mycorrhizal” functional group. None of the understory herbaceous species in our study formed mycorrhizal associations with non‐AM fungi (i.e., ectomycorrhizal, ericoid). We excluded species from our analyses with unknown mycorrhizal status. The relative abundance of species with an unknown mycorrhizal status averaged 1.6% of the total abundance. Since we manipulated garlic mustard abundance by weeding one‐half of each plot, garlic mustard was also excluded from all analyses. In total, we were able to evaluate garlic mustard’s effects on 28 plant taxa (19 mycorrhizal, 9 non‐mycorrhizal) across our experimental plots.

We characterized shifts in mycorrhizal and non‐mycorrhizal components of the plant community by quantifying changes in community dissimilarity in response to garlic mustard weeding and year. We further described patterns of community change by analyzing the effects of garlic mustard weeding and year on species richness, evenness, and diversity of mycorrhizal and non‐mycorrhizal plant species. We also tested for a difference in the abundance of mycorrhizal and non‐mycorrhizal plants between the garlic mustard ambient and weeded treatments. Finally, we asked whether individual species showed consistent responses that could be predicted by their functional group (i.e., mycorrhizal status).

### Community composition

We tested for the effects of garlic mustard on community composition for the mycorrhizal and the non‐mycorrhizal plant communities separately because we expected only mycorrhizal plant species to directly respond to garlic mustard removal. We predicted that weeding would affect mycorrhizal plant communities, with increasing dissimilarity between garlic mustard ambient and weeded treatments over time, whereas we did not expect differences between treatments for non‐mycorrhizal plant communities. PERMANOVA is based on the ratio of differences in dissimilarity between vs. within groups (Anderson 2014), which we used to compare dissimilarity in composition between ambient and garlic mustard weeded treatments and to test for different trends over time within those treatments. For each plant functional group, we used a PERMANOVA with an interaction between garlic mustard treatment and year of sampling to explain Bray‐Curtis dissimilarities between plant communities in the garlic mustard ambient and garlic mustard weeded treatments. The permutations were constrained within garlic mustard treatment nested within plot nested within year.

### Diversity

To determine whether garlic mustard weeding changed diversity in the plots over time, we calculated species richness, Pielou’s evenness, Shannon’s diversity, and inverse Simpson diversity of the mycorrhizal plant community and of the non‐mycorrhizal plant community every year for each treatment in each plot. Of the two diversity indices (Shannon and inverse Simpson), Shannon’s diversity is more sensitive to changes in rare species, whereas inverse Simpson diversity is more sensitive to changes in highly abundant species (Morris et al. 2014). We included all of these metrics because compound measurements such as Shannon and inverse Simpson diversity contain slightly different information about which piece of diversity is changing (rare or abundant species). In addition, Pielou’s evenness tends to correlate highly with Shannon’s diversity, so it is useful to interpret both of these indices alongside inverse Simpson diversity, because it is independent from evenness and Shannon’s diversity (Morris et al. 2014). For each of these four diversity metrics, we fit a linear model with garlic mustard treatment, year, and their interaction as fixed effects. We included plot as a random effect to account for repeated measurements of each plot across years.

### Abundance

We tested for changes in abundance within each functional group (i.e., mycorrhizal status) between garlic mustard ambient and weeded treatments using a generalized linear mixed effects model including the garlic mustard treatment, year, and their interaction as fixed effects. We fit separate models for each functional group, with the summed percent cover of all mycorrhizal species or of all non‐mycorrhizal species, as the response variable, respectively. To control for non‐independence due to repeated sampling of plots over time, we included plot as a random effect. Summed percent cover for each functional group never exceeded 100% so we modeled proportional cover using a beta distribution.

### Individual species response

We tested whether mycorrhizal status affected species‐specific responses to garlic mustard treatments. To do this, we used the Relative Interaction Index (RII; Armas et al. 2004), which calculates the biomass response to a biological interaction, here, the relative biomass difference with and without garlic mustard

$$\text{RII}=\frac{B_{a}‐B_{w}}{B_{a}+B_{w}}.$$

*B*_w_ in our study is the abundance of the focal species in the garlic mustard weeded treatment, and *B*_a_ is abundance in the ambient treatment within the same plot. Therefore, any negative RII values (i.e., *B*_w_> *B*_a_) indicate a negative effect of garlic mustard on abundance, while positive RII values indicate a positive effect. To assess differences in RII, we fit a general linear mixed effects model including the garlic mustard treatment, year, and their interaction as fixed effects and plant species and plot as random effects. We predicted that mycorrhizal plant species would have more negative RII values, as well as a more negative temporal trend in RII than non‐mycorrhizal plant species. RII is only a meaningful metric when abundance is greater than zero in both the presence and absence of the biological interaction, so we only calculated RII when the focal species was present in both treatments for a plot and year (16 mycorrhizal species and 6 non‐mycorrhizal species).

Native plant response to garlic mustard invasion may be dependent on evolutionary history (e.g., have a phylogenetic signal). We tested for a phylogenetic signal (*K*; Blomberg et al. 2003) in all metrics of native plant response separately for each year and treatment on an ultrametric super‐tree phylogeny created following methods in Worchel et al. (2013). After correcting for multiple comparisons with a Bonferroni correction, there was no consistent effect of phylogenetic history of native plant responses to invasion and therefore we did not account for phylogenetic history in our statistical analyses.

All statistical analyses were conducted in R version 3.5.1 (R Core Team 2018). We tested for phylogenetic signal using the Picante package version 1.8 (Kembel et al. 2010). Linear models were estimated with the glmmTMB package version 0.2.2.0 (Brooks et al. 2017). We assessed the fit of these models using the DHARMa package version 0.2.0 (Hartig 2016). Treatment effects of the linear models were tested using a Wald chi‐squared test. We analyzed linear model interaction terms using Type III sum of squares and, when interactions were not significant, we analyzed main effects using Type II sum of squares. We tested for significant pairwise comparisons between garlic mustard treatments within year at *P* = 0.05 using the emmeans package version 1.3.5.1 (Lenth 2019). We used the vegan package version 2.5‐3 (Oksanen et al. 2019) to calculate diversity metrics and to test for changes in community composition using PERMANOVA.

## Results

All analyses were performed with data from years 2006 to 2016. There were no significant differences in composition, diversity or abundance of the mycorrhizal or non‐mycorrhizal plant communities prior to establishment of the garlic mustard ambient and weeded treatments in 2006. These results suggest that the garlic mustard weeded treatment drove any subsequent changes in the mycorrhizal and non‐mycorrhizal plant communities. Since we manipulated the presence of garlic mustard as part of the experimental design, data on garlic mustard abundance were excluded from all analyses. We report the test statistics for the main effects between the garlic mustard treatment and year in Appendix S1: Table S2 for the PERMANOVA, and in Appendix S1: Table S3 for the linear models. All significant differences are for *P* ≤ 0.05.

### Community composition

**Fig. 2:**
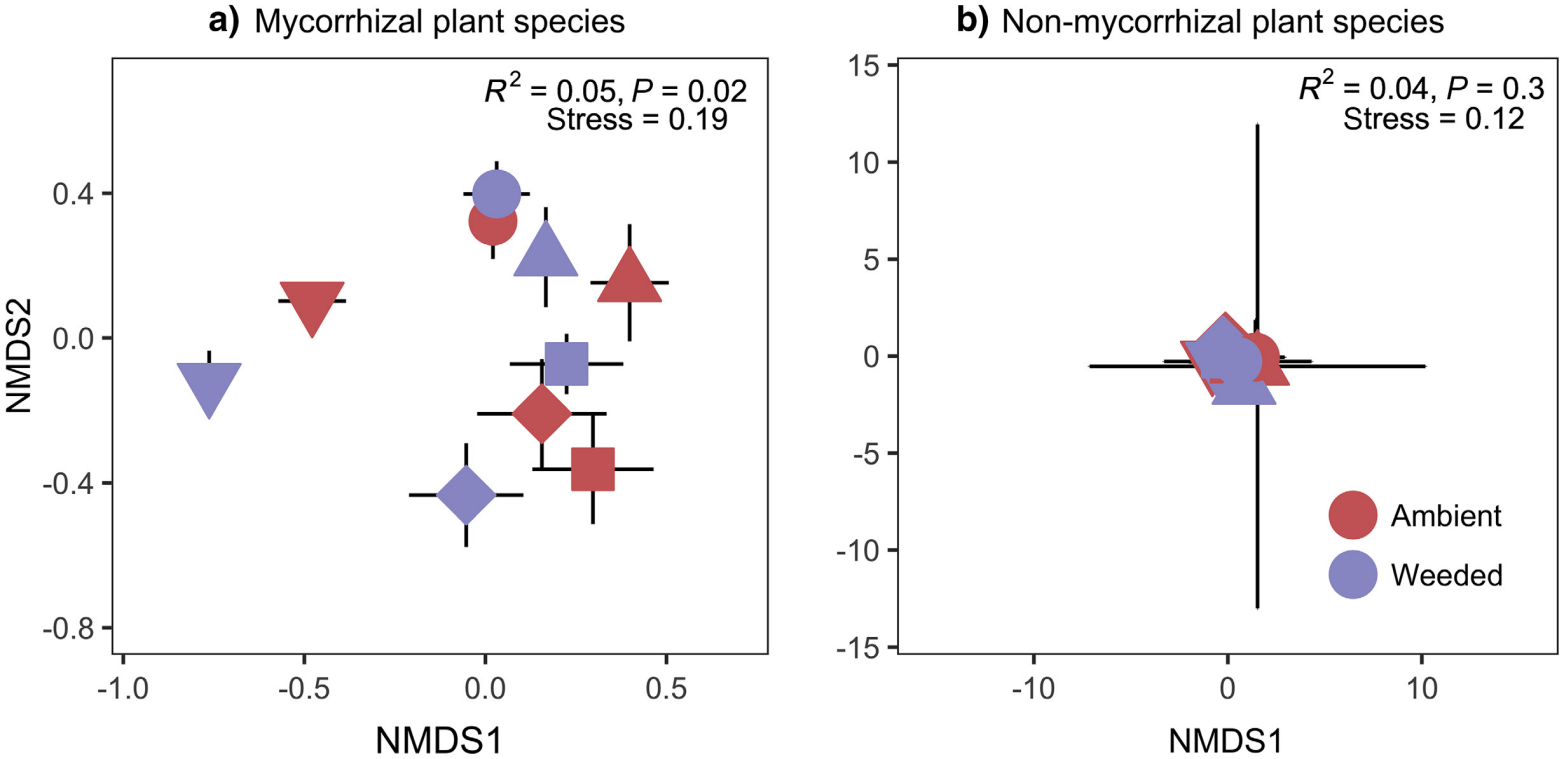
Ordination plots of Bray‐Curtis dissimilarities illustrating differences in composition between garlic mustard ambient (red) and garlic mustard weeded (purple) treatments for (a) mycorrhizal and (b) non‐mycorrhizal plant communities. Shapes represent different plots, plot 1, down‐pointing triangle; plot 2, square; plot 3, diamond; plot 4, up‐pointing triangle; plot 5, circle. Symbols are plot means with 95% confidence intervals for data collected every other year over 11 yr (2006–2016). Garlic mustard data was excluded from PERMANOVA and ordinations. Mycorrhizal plant communities differ significantly between treatments while non‐mycorrhizal plant communities did not.

Composition of the mycorrhizal plant community (Fig. 2a) differed between the garlic mustard ambient and garlic mustard weeded treatments. Conversely, the non‐mycorrhizal plant community did not differ significantly between treatments (Fig. 2b). Community composition of both functional groups (mycorrhizal and non‐mycorrhizal) also changed over time (Appendix S1: Fig. S1), However, we found no significant interaction between community composition and year in either functional group.

### Diversity

**Fig. 3:**
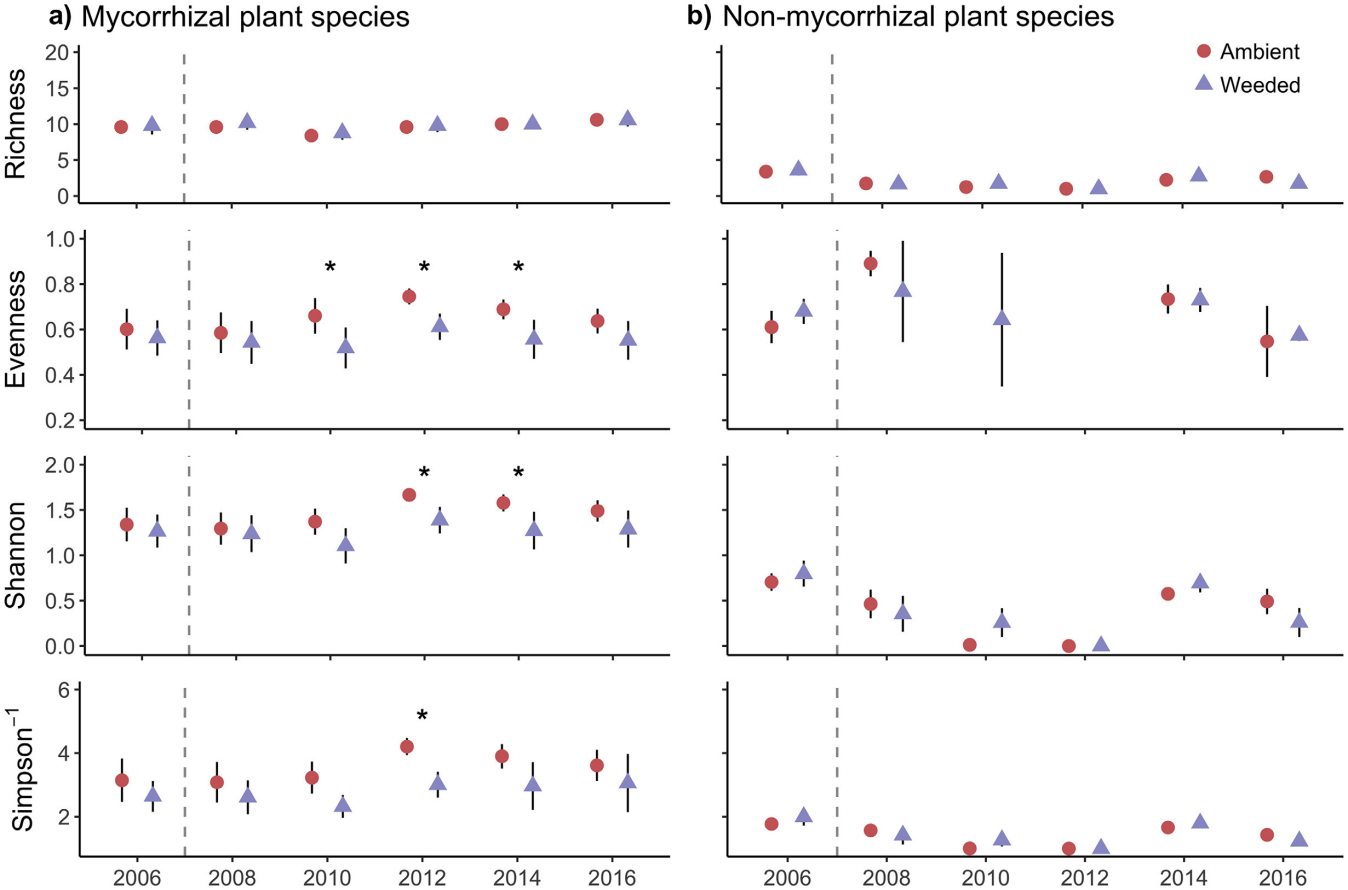
Species richness, evenness, Shannon diversity, and inverse Simpson (Simpson^−1^) diversity for (a) mycorrhizal plant species and (b) non‐mycorrhizal plant species. Points represent plot means for garlic mustard ambient (red circles) and garlic mustard weeded (purple triangles) treatments (2006–2016). Garlic mustard data were excluded from calculation of diversity metrics. Error bars are ±SE. Vertical dashed lines separate pre‐garlic mustard weeded (2006) from post‐garlic mustard weeded time points. The interaction and main effects of garlic mustard treatment and time were nonsignificant for species richness for both mycorrhizal and non‐mycorrhizal plant species. The main effect of garlic mustard treatment was significantly different for evenness, Shannon and inverse Simpson diversity for mycorrhizal plant species, but not the non‐mycorrhizal plant species. Asterisks indicate significant pairwise comparisons at *P* = 0.05.

Garlic mustard weeding changed the diversity of mycorrhizal but not non‐mycorrhizal functional group. There was no effect of garlic mustard treatment or year on species richness of mycorrhizal plants (Fig. 3a). Richness of non‐mycorrhizal plants did not respond to garlic mustard treatment but changed over time (Fig. 3b). Evenness of the mycorrhizal functional group overall was 15.2% lower in the garlic mustard weeded treatments but did not change over time (Fig. 3a). In contrast, evenness of the non‐mycorrhizal functional group did not change between garlic mustard treatments but did change over time (Fig. 3b). Shannon and Inverse Simpson diversity of the mycorrhizal group decreased overall by 14% and 23%, respectively, in garlic mustard weeded treatments (Fig. 3a), but garlic mustard treatment had no effect on Shannon or inverse Simpson diversity of non‐mycorrhizal group (Fig. 3b). There was no temporal trend in Shannon or inverse Simpson diversity for the mycorrhizal functional group, but there was a significant effect of year on Shannon and inverse Simpson diversity for the non‐mycorrhizal functional group.

### Abundance

**Fig. 4:**
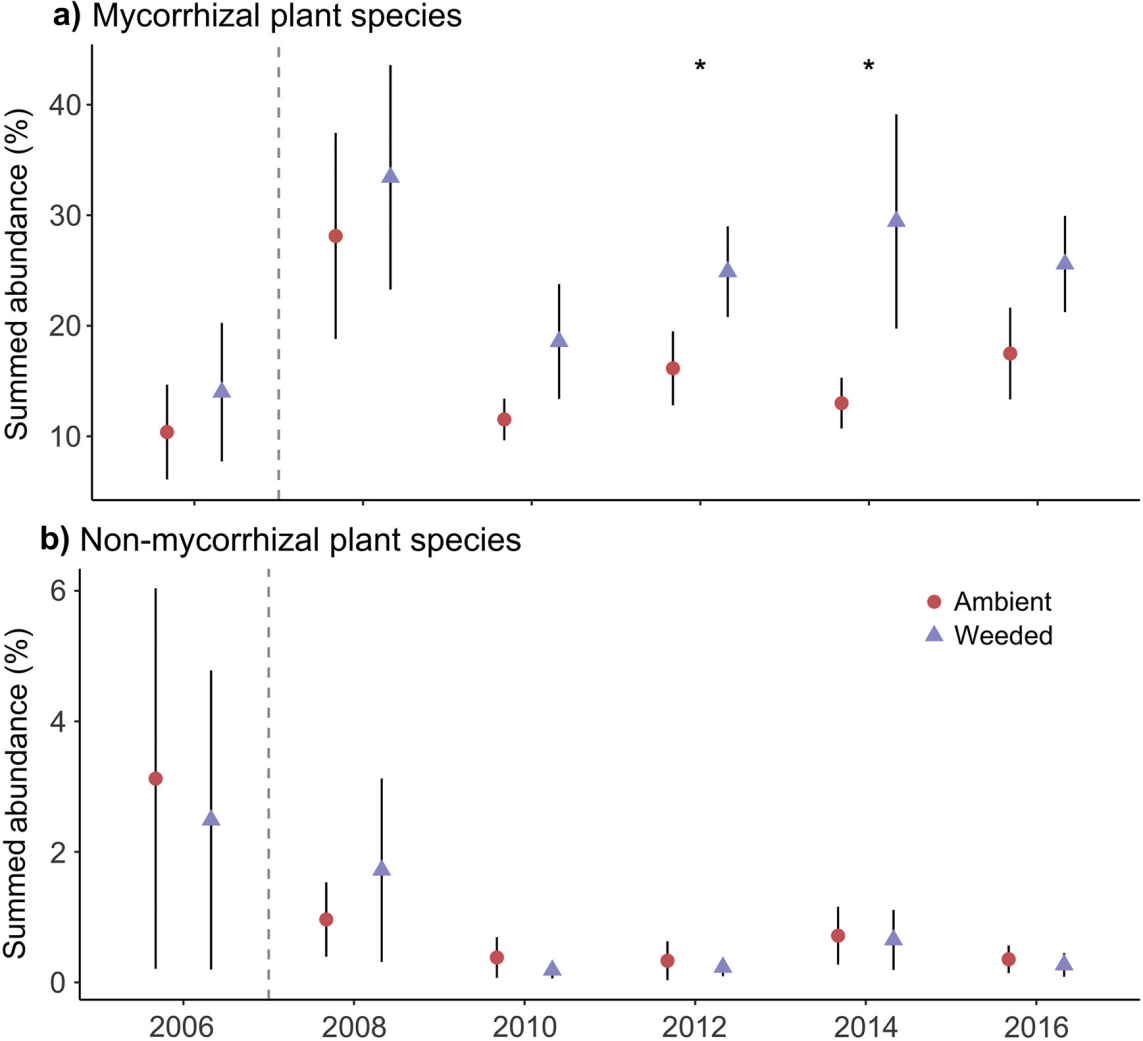
Effect of garlic mustard treatment over time on summed abundance of (a) mycorrhizal plant species and (b) non‐mycorrhizal plant species. Points are plot means of summed abundance in garlic mustard ambient (red circles) and garlic mustard weeded (purple triangles) treatments (2006–2016). Garlic mustard abundance was excluded. Error bars are ±SE. Vertical dashed lines separate pre‐garlic mustard weeded (2006) from post‐garlic mustard weeded time points. Summed abundance between treatments was not significantly different in 2006 (pre‐garlic mustard weeding). There was a significant main effect of the garlic mustard treatment on summed abundance of mycorrhizal plants, but no significant interaction between garlic mustard treatment and time. For non‐mycorrhizal plant species, the main effect of treatment was not significant, while the main effect of time was significant; there was no significant interaction between garlic mustard treatment and time. Asterisks indicate significant pairwise comparisons at *P* = 0.05.

Weeding garlic mustard from the plots resulted in an overall 19.7% increase in abundance of mycorrhizal plant species relative to those in the ambient treatment (Fig. 4a). In contrast, there was no change in abundance of non‐mycorrhizal species between garlic mustard treatments (Fig. 4b). The overall abundance of both mycorrhizal and non‐mycorrhizal species changed over time, but these changes were independent of garlic mustard weeding. There was no significant interaction between garlic mustard treatment and year for abundance of mycorrhizal or non‐mycorrhizal species.

### Individual species response

**Fig. 5:**
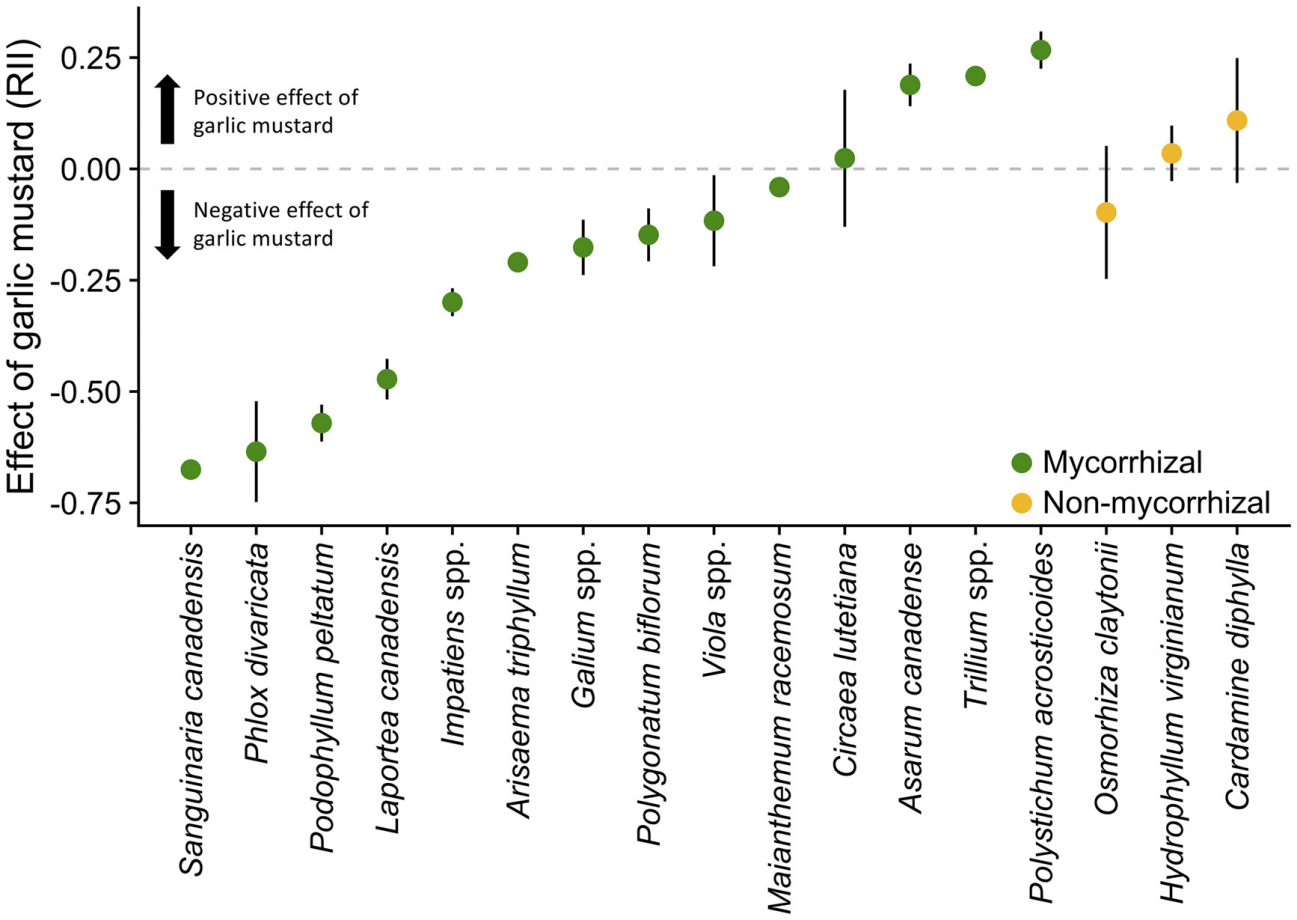
Relative interaction index (RII) of each plant species between garlic mustard ambient and garlic mustard weeded treatments. A negative RII indicates that a species relative abundance is higher in the weeded treatment than in the ambient treatment. Conversely, a positive RII indicates that a species has lower relative abundance in the ambient treatment. Points are the mean RII for each species across plots and years, error bars are ±SE.

Within functional group (mycorrhizal status), there was variation in strength and direction of each plant species RII response (Fig. 5). Ten mycorrhizal plant species increased in abundance in garlic mustard weeded vs. ambient treatments resulting in a negative RII, whereas four mycorrhizal species had a positive RII. One non‐mycorrhizal plant species had a higher abundance in garlic mustard weeded vs. ambient plots whereas two non‐mycorrhizal species had a positive RII. There was no effect of year or mycorrhizal status on the effect of garlic mustard (RII).

## Discussion

Numerous studies demonstrate that invasive plants have negative impacts on native species (Vilà et al. 2011, Pyšek et al. 2012). However, to anticipate *how* resident plant communities shift and which native species are most likely to be impacted by an invader, we must explore the mechanisms by which invasive plants displace natives (Levine et al. 2003, Sofaer et al. 2018). Using detailed data from a 11‐yr manipulative field experiment, we found that the removal of garlic mustard (i.e., weeded treatment) altered the composition, decreased the diversity, and increased the abundance of the mycorrhizal plant community in comparison to ambient levels of garlic mustard invasion. In contrast, there were no effects of the garlic mustard treatments on the non‐mycorrhizal plant community, which is not susceptible to garlic mustard’s disruption of mycorrhizal mutualisms. Our study illustrates that the consequences of garlic mustard invasion for the plant community may be anticipated by incorporating an understanding of mutualism disruption into future invasion predictions, because garlic mustard weeding favored mycorrhizal, but not non‐mycorrhizal plant species. Our results are consistent with previous work that found variation in response to garlic mustard by mycorrhizal dependency of forest tree seedlings (Stinson et al. 2006). Together, our findings and previous work demonstrate that garlic mustard invasion results in a functional shift in plant community membership that can be reversed over time with removal of the invader.

The shifts in the composition, diversity, and abundance of the mycorrhizal, but not the non‐mycorrhizal, functional group in response to garlic mustard weeding are indicative of mutualism disruption. Other mechanisms of impact, such as direct competition or direct phytotoxicity, would not disproportionately affect species that form mycorrhizal associations. Garlic mustard is a poor direct competitor in its invasive range (Meekins and McCarthy 1999, Bossdorf et al. 2004), and disrupts mycorrhizal mutualisms to indirectly compete against other plants (Brouwer et al. 2015, Hale et al. 2016). Removal of a competitive dominant plant species from a community is expected to result in an increase in abundance of other community members (MacDougall and Turkington 2005), but unlike mutualism disruption, exploitative competition would not explicitly predict different responses for mycorrhizal and non‐mycorrhizal plants to the removal of a competitor. Since mycorrhizal and non‐mycorrhizal plants differ in their methods of resource acquisition (via roots alone or via fungal partners), they may respond differently to limiting resources under exploitative competition. However, soil resources do not differ between garlic mustard ambient and weeded treatments at our site (Bialic‐Murphy, L. et al. *unpublished manuscript*) and are, therefore, unlikely to cause the trends we observed here. Observed patterns of the plant community response to garlic mustard in our field site are consistent with those predicted by the mechanistic process of mutualism disruption.

Previous work has quantified responses of root fungal communities to garlic mustard and provides context for interpreting our results. At our study site, root fungal communities within some species’ roots changed in response to our garlic mustard treatments (Burke 2008), particularly in the presence of deer (Burke et al. 2019). Ongoing work shows that species in the ambient garlic mustard treatments have reduced hyphal colonization in their roots, and higher evenness in the soil AM fungal community (Bialic‐Murphy, L. et al. *unpublished manuscript*). In other locations, studies have documented no recovery of mycorrhizal fungi after three years (Anthony et al. 2019) or only partial recovery after six years (Lankau et al. 2014). Since we did not observe a response in the aboveground community until 4–6 yr of garlic mustard weeding, it is possible that the aboveground response is mirroring the recovery of the belowground mycorrhizal fungal community, or that the mycorrhizal fungal community has not yet responded to garlic mustard removal. In addition, it is possible that changes in the abiotic soil conditions that are associated with garlic mustard invasion attribute to the observed response of mycorrhizal plant species (Rodgers et al. 2008, Anthony et al. 2019). However, no differences in abiotic soil conditions between ambient and weeded garlic mustard treatments after 14 yr were found by Bialic‐Murphy, L. et al. (*unpublished manuscript*). Thus, findings from our experiment provide a link between mechanistic evidence of mutualism disruption from careful fine‐scale experiments (e.g., Stinson et al. 2006, Hale et al. 2011, 2016, Brouwer et al. 2015) to observed shifts in mycorrhizal plant communities.

Our findings support the utility of our coarse classification into mycorrhizal and non‐mycorrhizal functional groups for anticipating responses to invasions but also highlight limitations in the degree of predictability within ecological communities. In particular, within the mycorrhizal group there was considerable variation in response to garlic mustard (RII) (Fig. 5), such that a simple binary assignment of mycorrhizal status would be insufficient to reliably predict changes in species‐level abundance. Plant species’ dependency on mycorrhizal partners (i.e., biomass response to mycorrhizal colonization) generally falls on a spectrum from highly positive plant biomass responses to mycorrhizal colonization to somewhat negative (Tawaraya 2003), and variation in dependence of mycorrhizal plants on their fungal partners is known to influence the response of a particular species to mutualism disruption (Stinson et al. 2006, McCary et al. 2019). We do not know where our naturally occurring plant species fall along the spectrum of dependency on their mycorrhizal partners, and it is possible that differences among trajectories of the experimental plots in response to garlic mustard weeding (Fig. 2a) arise from variation in the dependency of the mycorrhizal species naturally occurring in those plots. Exploring the factors that may have driven the different trajectories of the unique plant communities in each plot is an important next step in understanding the intricacies of community responses to garlic mustard invasion.

Our results revealed that the mycorrhizal plant community had decreased evenness and diversity in the garlic mustard weeded treatments. The decreases in Shannon and inverse Simpson diversity of the mycorrhizal plant community are likely driven by the lower evenness in the garlic mustard weeded treatments, since there was no change in species richness for this group. Evenness of the mycorrhizal plant community decreased in the garlic mustard weeded plots because of the dramatic increase in the abundance of a common annual species, *Impatiens* spp., after 2006 (Appendix S1: Fig. S2). This created a less balanced community with low evenness where one species (*Impatiens* spp.) has a very high abundance whereas the rest of the species remain at a low abundance. Since *Impatiens spp*. have an annual life history and often exhibit ruderal growth patterns in comparison to many of the long‐lived perennials at our site, we expect that the long‐lived perennial species will rebound as well with more time.

Over the course of this study, we found that the main effect of time on the abundance of both plant communities was significant, but the interactions between the garlic mustard treatment and year were not. The non‐mycorrhizal plant abundance decreased over time in both garlic mustard treatments. Therefore, this decline in non‐mycorrhizal plant abundance cannot be attributable only to the increase in mycorrhizal plant abundance in the garlic mustard weeded treatment. We see similar declines in non‐mycorrhizal plant abundance in other plots at our site that are not protected from deer herbivory by fences (*unpublished data*), indicating that exclusion of deer is also not driving the declines in non‐mycorrhizal plant abundance. These results suggest that there are other sources of environmental variability (e.g., annual fluctuations in precipitation patterns) that likely influenced the abundance of both plant community types over time. Despite this, there is a strong signature of the effects of garlic mustard weeding on mycorrhizal plant abundance.

Garlic mustard consistently has negative effects on native mycorrhizal plant species (e.g., Stinson et al. 2006, Cipollini et al. 2008, Wolfe et al. 2008). These negative effects on mycorrhizal plant species are consistent with garlic mustard’s disruption of native mycorrhizal mutualisms, which leads to water stress and carbon stress (Brouwer et al. 2015, Hale et al. 2016) and decreases in vital rates (Brouwer et al. 2015) and population growth rates (Bialic‐Murphy et al. 2019). In addition, garlic mustard alters plant communities (Stinson et al. 2007). Our results build on these findings to show that the outcomes symptomatic of mutualism disruption in individual plants follow a similar pattern for plant communities in the field. Our 11‐yr long‐term field experiment shows that, even at the modest garlic mustard abundances at Trillium Trail, mutualism disruption alters the community composition, changes diversity, and reduces the abundance of only the mycorrhizal plants within the entire plant community. We stress that the length of our experiment provides strong support for the long‐term effects of garlic mustard on the mycorrhizal plant community. Most studies that evaluate the impacts of invasive species last only 1 yr (Stricker et al. 2015) even though the effects of invaders are known to lag after the invasion establishes and have legacy effects after they are removed. Land managers attempting to restore native understories through garlic mustard removal may expect to see an increase in abundance of mycorrhizal plant species, but likely no changes in species richness. However, responses may differ by species’ sensitivity to garlic mustard (see Fig. 5 and Appendix S1: Fig. S3), and may require multiple years (in our experimental study 4–6 yr) of annual garlic mustard removal to occur.

Many invasive plant species are thought to impact native species by allelopathy and/or mutualism disruption (e.g., *Alliaria petiolata, Tamarisk* spp., various *Fallopia* species; Stinson et al. 2006, Wolfe et al. 2008, Bainard et al. 2009, Murrell et al. 2011, Hale and Kalisz 2012, Meinhardt and Gehring 2012), so our findings likely apply to the impacts of invasive plants beyond garlic mustard. In addition, since approximately 80% of understory herbs globally (and two‐thirds of the study species in our experimental plots; Appendix S1: Table S1) form mycorrhizal associations (Soudzilovskaia et al. 2020), there is potential for garlic mustard to drastically shift understory plant communities across its invasive range. We demonstrate that mycorrhizal mutualism disruption by an invasive plant leads to a shift in plant communities but found that responses were variable among species within functional groups. This suggests that the ability to predict invader impacts across space and time may require refinement of the focal functional trait, in this case a shift from a binary to a continuous view of mycorrhizal dependency. Our study highlights the utility of exploring the mechanisms by which invasive species cause impact because we find a shift in functional groups of the resident plant community that would only be anticipated by knowing that mechanism.

## Supporting information

Appendix S1

## Data Availability

Data are available from the USGS ScienceBase repository: https://doi.org/10.5066/P9VP7BFU
