# Supplementary material for: Negative effects of an allelopathic invader on AM fungal plant species drive community‐level responses
Source: Ecology. 2020 Nov 4;102(1):e03201. doi: 10.1002/ecy.3201 (PMC7816256; doi:10.1002/ecy.3201)
Supplement: Supplementary file 1 — Appendix S1 [file ECY-102-e03201-s001.pdf]

Supporting Information. Roche, M.D., I.S. Pearse, L. Bialic-Murphy, S.N. Kivlin, H.R. Sofaer, and S. Kalisz. 2020. Negative effects of an allelopathic invader on AM fungal plant species drive community-level responses. Ecology.

Appendix S1

Table S1. Plant species present in experimental plots at Trillium Trail Nature Reserve and their mycorrhizal status (19 mycorrhizal and 9 non-mycorrhizal).

Family	Scientific name	Common name	Mycorrhizal status	Citation
Amaryllidaceae	*Allium tricoccum*	wild leek	mycorrhizal	Brundrett and Kendrick 1988
Apiaceae	*Osmorhiza longistylis*	aniseroot	mycorrhizal	Trapp unpublished data
Apiaceae	*Osmorhiza claytonii*	hairy sweet-cicely	non-mycorrhizal	Trapp unpublished data
Araceae	*Arisaema triphyllum*	Jack-in-the-pulpit	mycorrhizal	Brundrett and Kendrick 1988
Aristolochiaceae	*Asarum canadense*	Canadian wild ginger	mycorrhizal	Brundrett and Kendrick 1988
Asparagaceae	*Maianthemum racemosum*	false Solomon's seal	mycorrhizal	Brundrett and Kendrick 1988
Asparagaceae	*Polygonatum biflorum*	Solomon's seal	mycorrhizal	Brundrett and Kendrick 1988
Balsaminaceae	*Impatiens spp.*(*capensis* and *pallida)*	touch-me-not	mycorrhizal	Brundrett and Kendrick 1988
Berberidaceae	*Podophyllum peltatum*	mayapple	mycorrhizal	Brundrett and Kendrick 1988
Boraginaceae	*Mertensia virginica*	Virginia bluebells	non-mycorrhizal	Trapp unpublished data
Brassicaceae	*Alliaria petiolata*	garlic mustard	non-mycorrhizal	Wang and Qiu 2006
Brassicaceae	*Cardamine concatenata*	cutleaf toothwort	non-mycorrhizal	Brundrett and Kendrick 1988
Brassicaceae	*Cardamine diphylla*	crinkleroot	non-mycorrhizal	Trapp unpublished data
Crassulaceae	*Sedum ternatum*	woodland stonecrop	non-mycorrhizal	Trapp unpublished data
Dryopteridaceae	*Polystichum acrostichoides*	Christmas fern	mycorrhizal	Kivlin unpublished data
Geraniaceae	*Geranium maculatum*	spotted geranium	mycorrhizal	Trapp unpublished data
Hydrophyllaceae	*Hydrophyllum virginianum*	Virginia waterleaf	non-mycorrhizal	Brundrett and Kendrick 1988
Limnanthaceae	*Floerkea proserpinacoides*	false mermaid	non-mycorrhizal	Demars 1996
Melanthiaceae	*Trillium spp.* (*grandiflora and erectum*)	trillium	mycorrhizal	Brundrett and Kendrick 1988
Montiaceae	*Claytonia virginica*	narrow-leaved spring beauty	non-mycorrhizal	Brundrett and Kendrick 1988
Onagraceae	*Circaea lutetiana*	broadleaf enchanter's nightshade	mycorrhizal	Brundrett and Kendrick 1988
Papaveraceae	*Sanguinaria canadensis*	bloodroot	mycorrhizal	Brundrett and Kendrick 1988
Phytolaccaceae	*Phytolacca americana*	pokeweed	non-mycorrhizal	Trapp unpublished data

Polemoniaceae	*Phlox divaricata*	wild blue phlox	mycorrhizal	Brundrett and Kendrick 1988
Ranunculaceae	*Thalictrum pubescens*	king of the meadow	mycorrhizal	Brundrett and Kendrick 1988
Rubiaceae	*Galium spp.*	bedstraw	mycorrhizal	Bueno et al. 2017
Urticaceae	*Laportea canadensis*	Canadian woodnettle	mycorrhizal	Brundrett and Kendrick 1988
Urticaceae	*Pilea pumila*	Canadian clearweed	mycorrhizal	Brundrett and Kendrick 1988
Violaceae	*Viola spp.*	violet	mycorrhizal	Brundrett and Kendrick 1988

Table S2. Table of PERMANOVA statistics for the main effects of garlic mustard treatment and year on species composition for both mycorrhizal and non-mycorrhizal functional groups.

	Mycorrhizal composition			Non-mycorrhizal composition		
	F-statistic	df	p	F-statistic	df	p
Garlic mustard treatment	3.127	1	0.017	1.161	1	0.313
Year	3.170	1	0.017	2.696	1	0.006

Table S3. Table of χ^2 test statistics for the main effects of garlic mustard treatment and year on species richness, Pielou's evenness, Shannon diversity, Inverse Simpson diversity, and summed abundance for both mycorrhizal and non-mycorrhizal functional groups, and the main effects of mycorrhizal status and year on RII.

	Mycorrhizal richness			Non-mycorrhizal richness		
	χ^2	df	p	χ^2	df	p
Garlic mustard treatment	0.868	1	0.352	0.149	1	0.699
Year	22.691	5	<0.001	102.540	5	<0.001
	Mycorrhizal evenness			Non-mycorrhizal evenness		
	χ^2	df	p	χ^2	df	p
Garlic mustard treatment	10.398	1	0.001	1.414	1	0.234
Year	6.277	5	0.280	19.023	4	0.001
	Mycorrhizal Shannon			Non-mycorrhizal Shannon		
	χ^2	df	p	χ^2	df	p
Garlic mustard treatment	9.618	1	0.002	0.282	1	0.596
Year	9.927	5	0.077	64.979	5	<0.001
	Mycorrhizal Inverse Simpson			Non-mycorrhizal Inverse Simpson		
	χ^2	df	p	χ^2	df	p
Garlic mustard treatment	12.514	1	<0.001	0.461	1	0.497
Year	8.936	5	0.112	39.451	5	<0.001
	Mycorrhizal summed abundance			Non-mycorrhizal summed abundance		
	χ^2	df	p	χ^2	df	p
Garlic mustard treatment	12.615	1	<0.001	0.110	1	0.740
Year	31.050	5	<0.001	96.477	5	<0.001
	RII					
	χ^2	df	p			
Mycorrhizal status	2.375	1	0.123			
Year	3.860	5	0.570			

Figure S1. Ordination plots of Bray-Curtis dissimilarities illustrating differences in plant community composition over time for a) and b) mycorrhizal and c) and d) non-mycorrhizal plant communities. For clarity, a) and c) garlic mustard ambient and b) and d) garlic mustard weeded treatments are plotted separately. Both mycorrhizal and non-mycorrhizal plant communities differ significantly between years. Arrows show direction of change from year to year. Shapes represent different plots, plot 1 = down pointing triangle, plot 2 = square, plot 3 = diamond, plot 4 = up pointing triangle, plot 5 = circle.

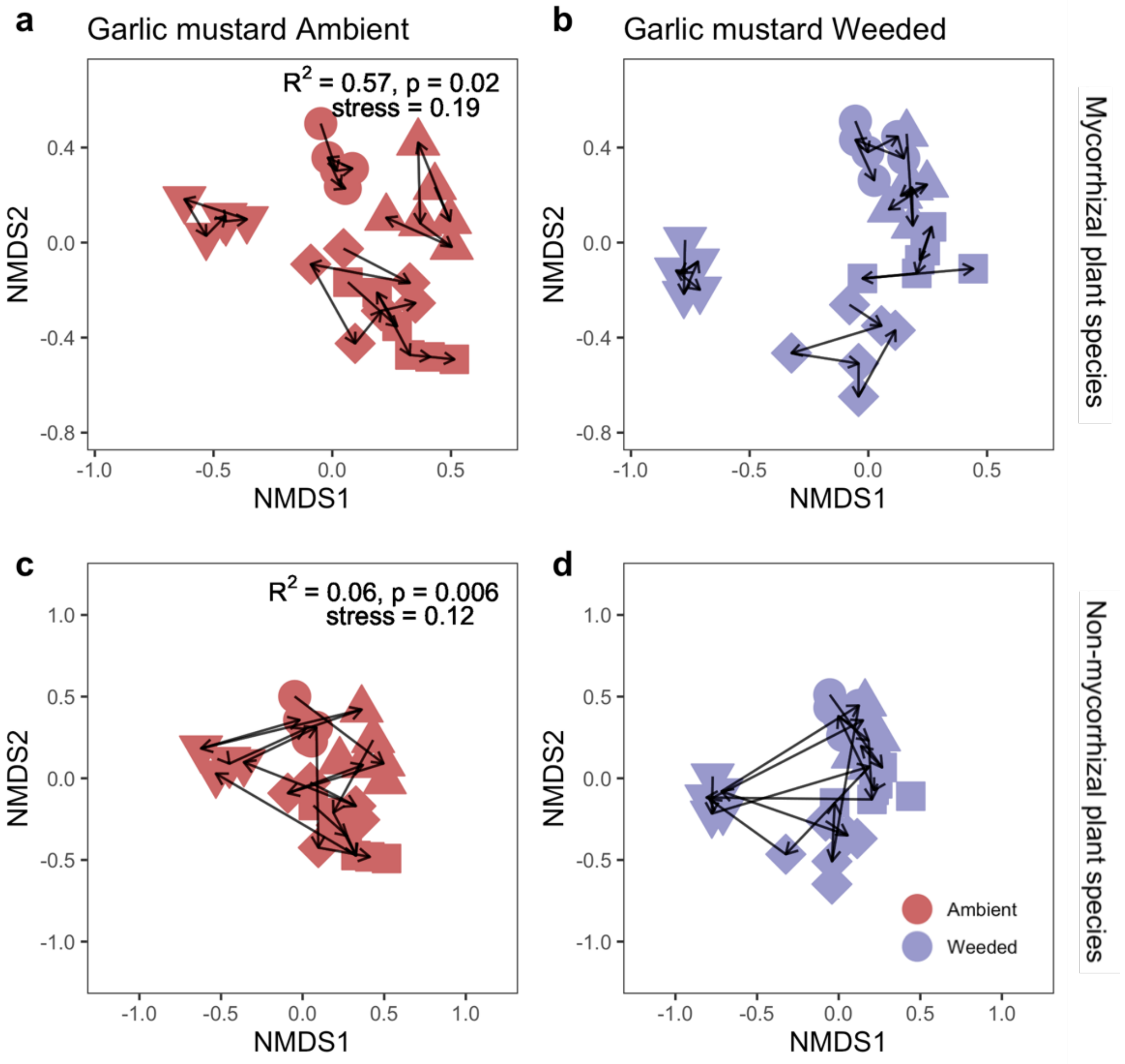

Figure S2. Rank abundance curves of a) mycorrhizal and b) non-mycorrhizal plant species in garlic mustard ambient and garlic mustard weeded treatments across the 14-year experiment. Species position in the 2006 rank order in garlic mustard ambient and weeded treatments was retained for all subsequent years. Points are plot means of relative abundance for each plant species in each year. Error bars are +/- 1SE. Rank abundance curves illustrate that shifts in diversity indices for mycorrhizal plant species are driven by an increase in dominance of *Impatiens spp.* in the weeded treatment. For non-mycorrhizal species, rank abundance curves show no distinguishable pattern between ambient and weeded treatments.

This should be 11 years, not 14

a. Mycorrhizal plant species

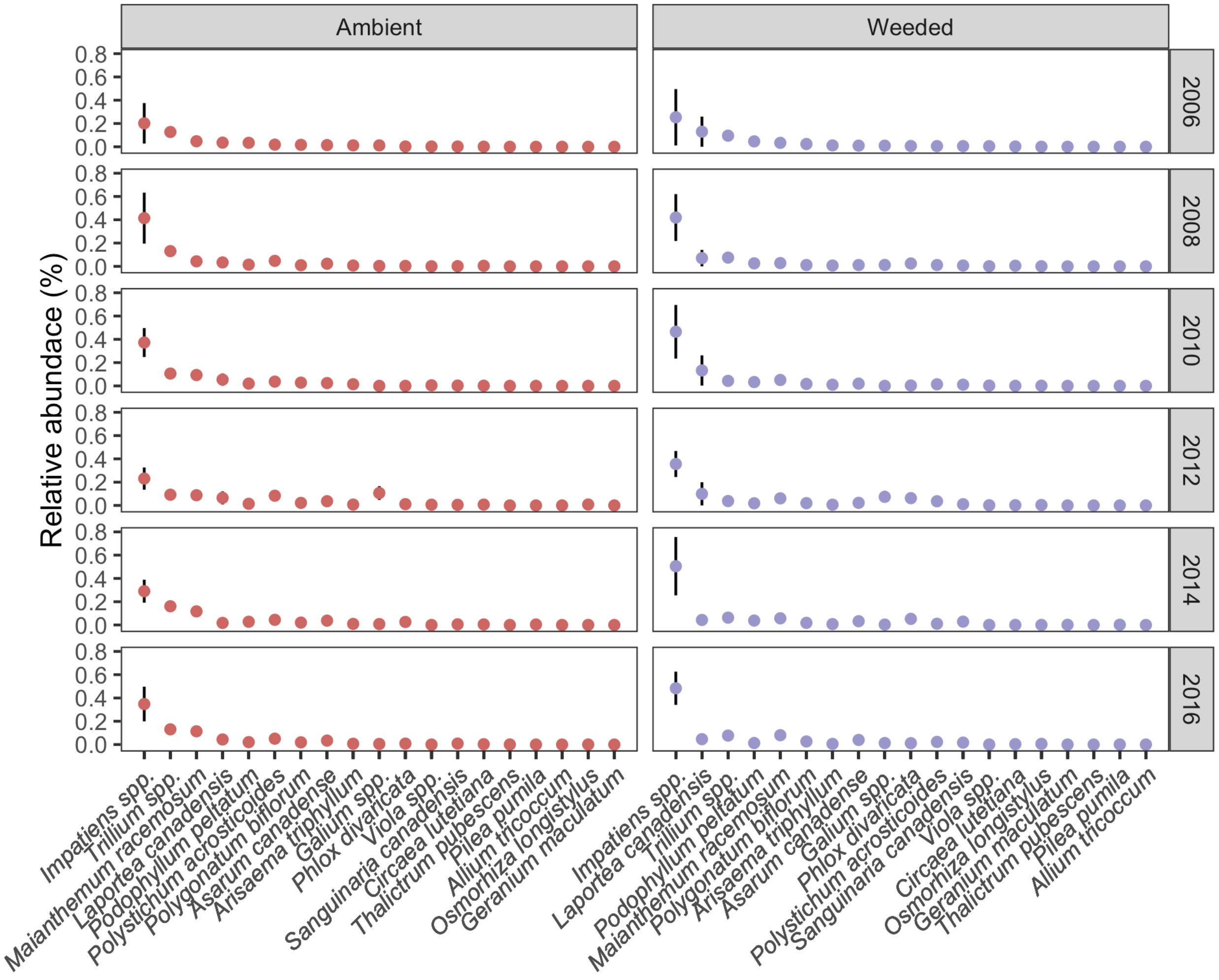

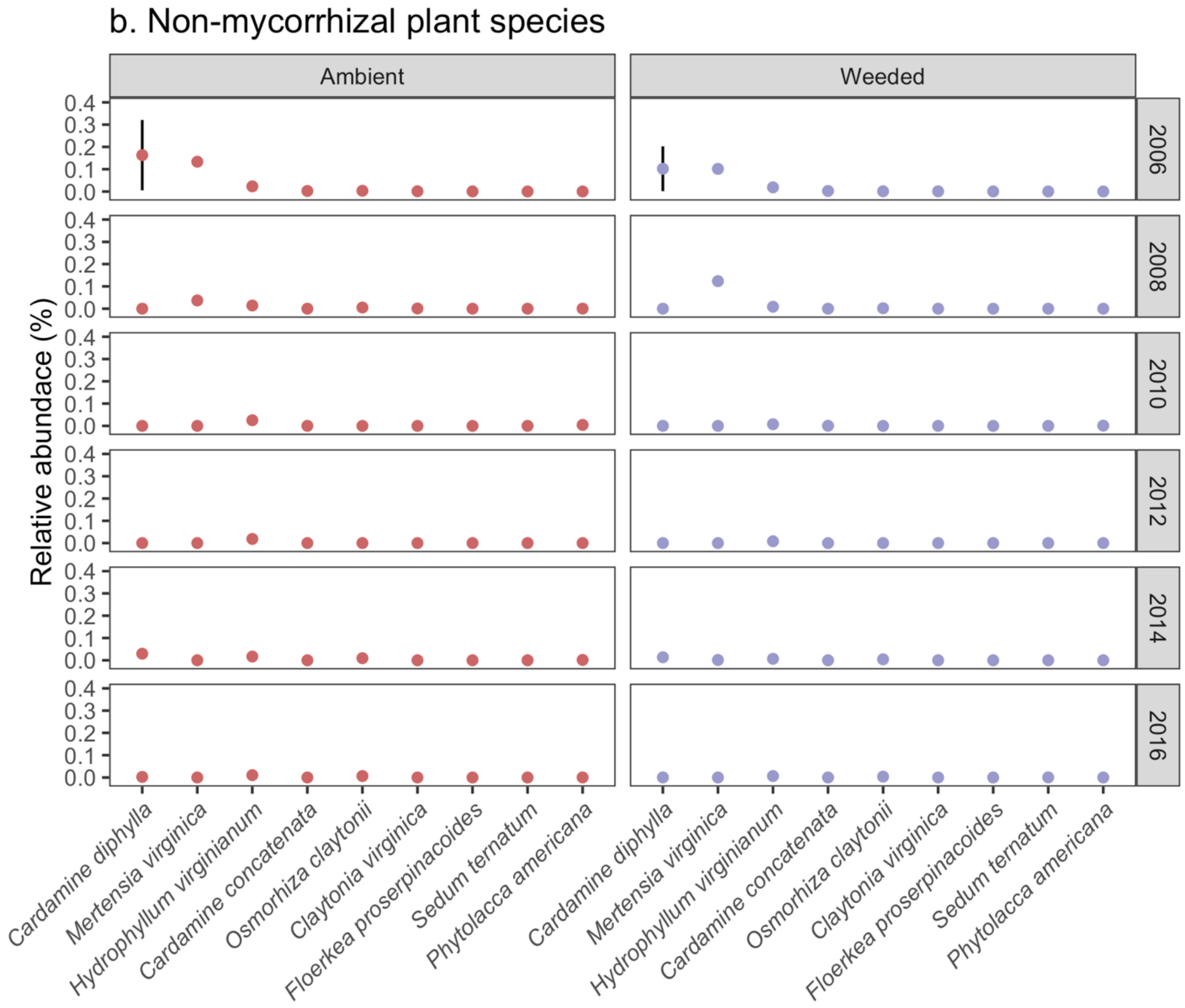

b. Non-mycorrhizal plant species
Ambient
Weeded
2006
2008
2010
2012
2014
2016
Relative abundace (%)
0.0
0.1
0.2
0.3
0.4
Cardamine diphylla
Mertensia virginica
Hydrophyllum virginianum
Cardamine concatenata
Osmorhiza claytonii
Claytonia virginica
Floerkea proserpinacoides
Sedum ternatum
Phytolacca americana

Figure S3. Relative interaction index (RII) of each plant species between garlic mustard ambient and garlic mustard weeded treatments for each year. A negative RII indicates that a species relative abundance is higher in the weeded treatment than in the ambient treatment. Conversely, a positive RII indicates that a species has lower relative abundance in the ambient treatment. Points are the mean RII for each species across plots, error bars are +/- 1 SE.

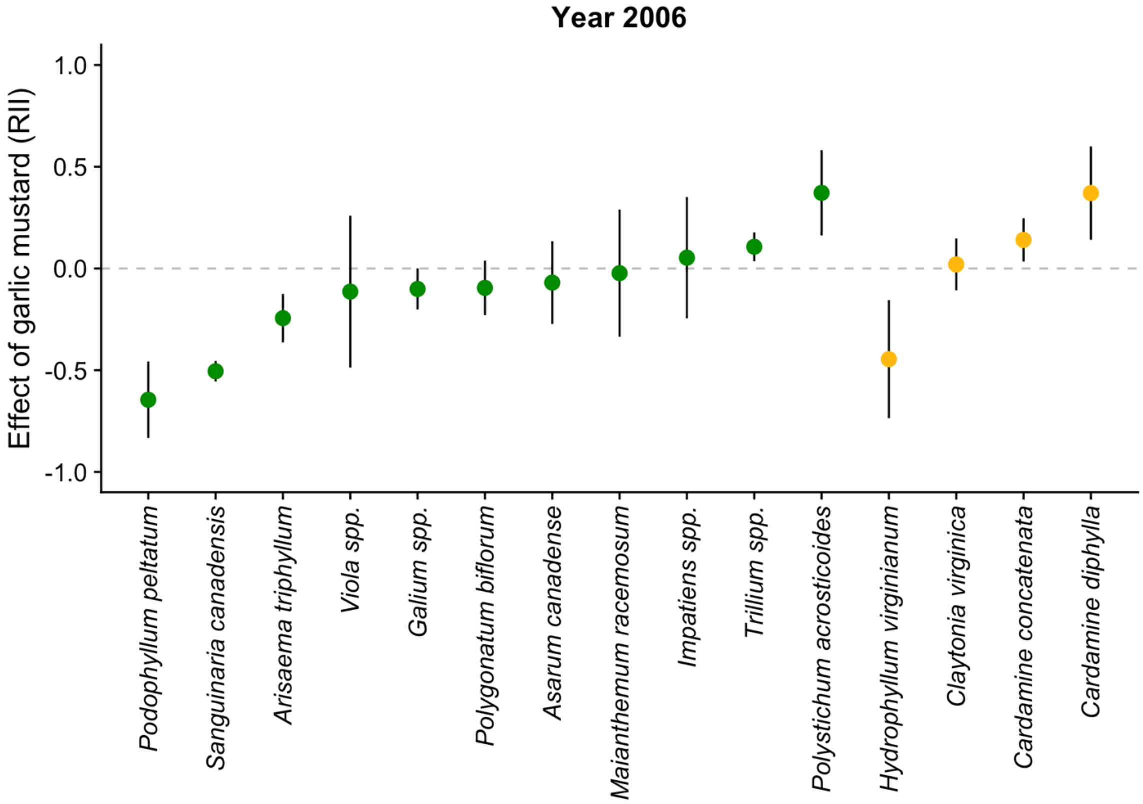

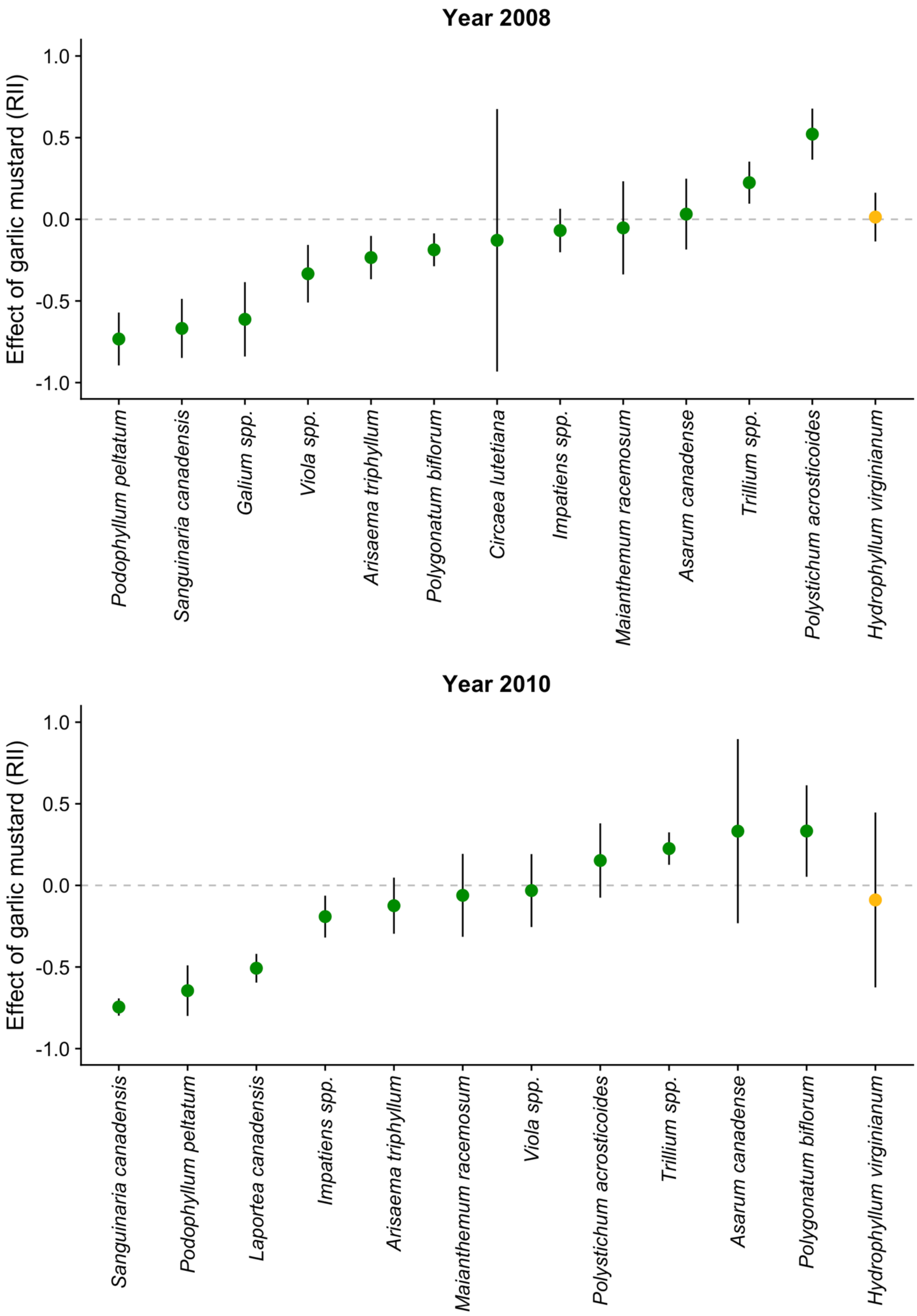

Year 2008
Effect of garlic mustard (RII)
1.0
0.5
0.0
-0.5
-1.0
Podophyllum peltatum
Sanguinaria canadensis
Galium spp.
Viola spp.
Arisaema triphyllum
Polygonatum biflorum
Circaea lutetiana
Impatiens spp.
Maianthemum racemosum
Asarum canadense
Trillium spp.
Polystichum acrosticoides
Hydrophyllum virginianum
Year 2010
Effect of garlic mustard (RII)
1.0
0.5
0.0
-0.5
-1.0
Sanguinaria canadensis
Podophyllum peltatum
Laportea canadensis
Impatiens spp.
Arisaema triphyllum
Maianthemum racemosum
Viola spp.
Polystichum acrosticoides
Trillium spp.
Asarum canadense
Polygonatum biflorum
Hydrophyllum virginianum

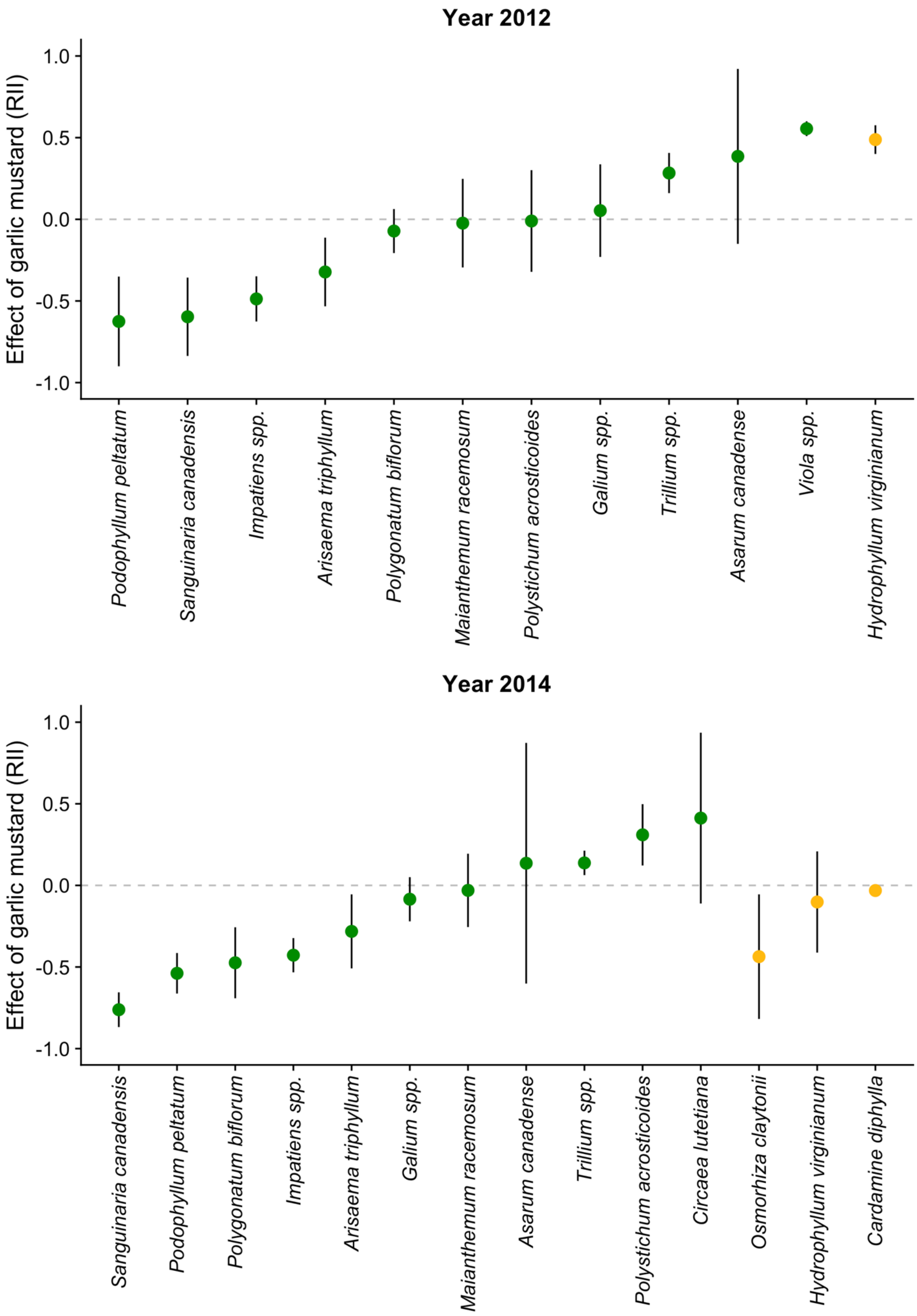
Year 2012
Effect of garlic mustard (RII)
1.0
0.5
0.0
-0.5
-1.0
Podophyllum peltatum
Sanguinaria canadensis
Impatiens spp.
Arisaema triphyllum
Polygonatum biflorum
Maianthemum racemosum
Polystichum acrosticoides
Galium spp.
Trillium spp.
Asarum canadense
Viola spp.
Hydrophyllum virginianum
Year 2014
Effect of garlic mustard (RII)
1.0
0.5
0.0
-0.5
-1.0
Sanguinaria canadensis
Podophyllum peltatum
Polygonatum biflorum
Impatiens spp.
Arisaema triphyllum
Galium spp.
Maianthemum racemosum
Asarum canadense
Trillium spp.
Polystichum acrosticoides
Circaea lutetiana
Osmorhiza claytonii
Hydrophyllum virginianum
Cardamine diphylla

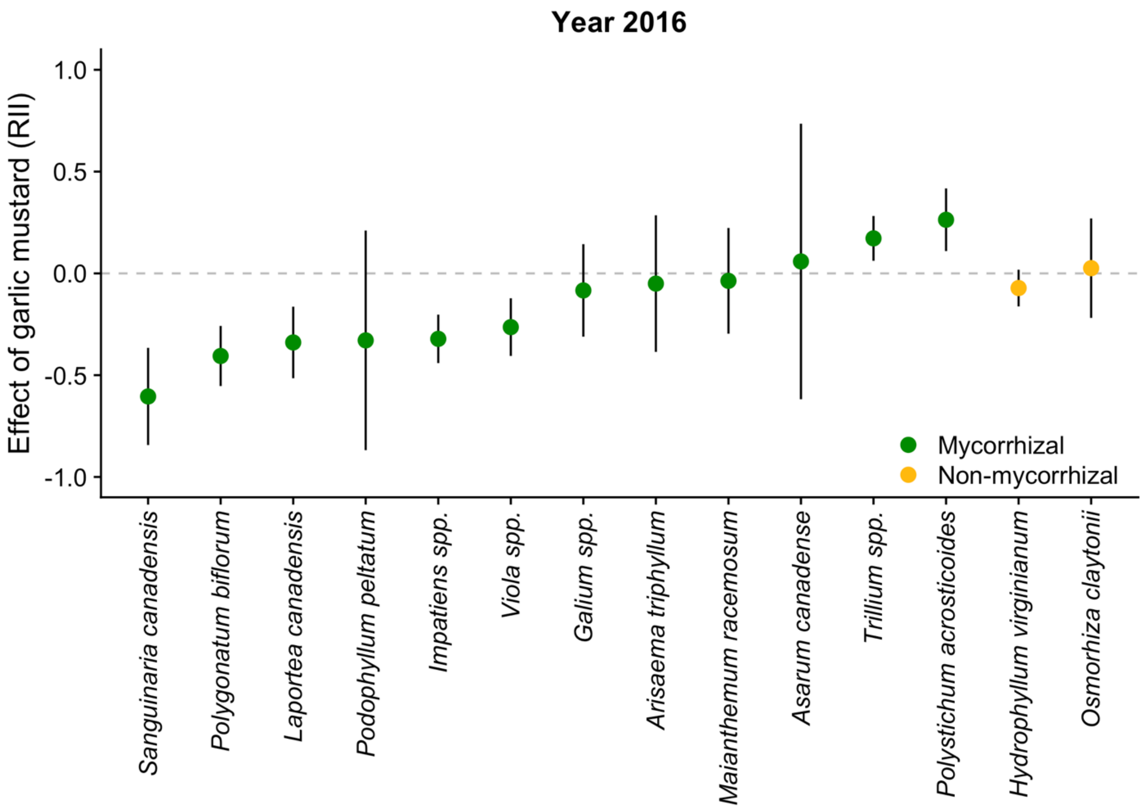

Year 2016
Effect of garlic mustard (RII)
1.0
0.5
0.0
-0.5
-1.0
Mycorrhizal
Non-mycorrhizal
Sanguinaria canadensis
Polygonatum biflorum
Laportea canadensis
Podophyllum peltatum
Impatiens spp.
Viola spp.
Galium spp.
Arisaema triphyllum
Maianthemum racemosum
Asarum canadense
Trillium spp.
Polystichum acrosticoides
Hydrophyllum virginianum
Osmorhiza claytonii

LITERATURE CITED

Brundrett, M. C., and B. Kendrick. 1988. The mycorrhizal status, root anatomy, and phenology of plants in a sugar maple forest. Canada Journal of Botany 66:1153–1173.

Bueno, C. G., M. Moora, M. Gerz, J. Davison, M. Öpik, M. Pärtel, A. Helm, A. Ronk, I. Kühn, and M. Zobel. 2017. Plant mycorrhizal status, but not type, shifts with latitude and elevation in Europe. Global Ecology and Biogeography 26:690–699.

Demars, B. G. 1996. Vesicular-arbuscular mycorrhizal status of spring ephemerals in two Ohio forests. Ohio Journal of Science 96:97–99.

Wang, B., and Y. L. Qiu. 2006. Phylogenetic distribution and evolution of mycorrhizas in land plants. Mycorrhiza 16:299–363.
